# The association between diabetes and hypertension with the number and extent of weight cycles determined from 6 million participants

**DOI:** 10.1038/s41598-022-09221-w

**Published:** 2022-03-28

**Authors:** Su Hwan Kim, Jin-Seul Kwak, Seong Pyo Kim, Sung Hyouk Choi, Hyung-Jin Yoon

**Affiliations:** 1 Biomedical Research Institute, Seoul National University Hospital, Seoul, Republic of Korea; 2 Department of Biomedical Engineering, Seoul National University College of Medicine, Seoul, Republic of Korea; 3 Interdisciplinary Program of Medical Informatics, Seoul National University College of Medicine, Seoul, Republic of Korea; 4 Medical Big Data Research Center, Seoul National University Medical Research Center, Seoul National University College of Medicine, Seoul, Republic of Korea

**Keywords:** Metabolism, Medical research, Diabetes, Risk factors, Diabetes, Obesity, Hypertension

## Abstract

The purpose of this study was to elucidate the association between weight cycling and clinical outcomes such as type 2 diabetes and hypertension with differential effects of baseline age and obesity. Nationwide data from 6,132,569 healthy adults who underwent five or more health screenings between 2002 and 2011 were analyzed and followed until December 2019 for type 2 diabetes and hypertension. Weight cycling was defined as a change in body weight followed by another change in the opposite direction. Through the Cox proportional hazards model, the number and degree of weight cycles were positively associated with increased risk of type 2 diabetes and hypertension. The hazard ratios (HRs) of weight cycling for type 2 diabetes and hypertension were as high as 1.263 (1.213–1.315) and 1.175 (1.144–1.207) at two or more weight cycles of 10% of body weight (BW), respectively. The association was stronger for females, individuals with normal body weight/BMI, and older individuals. Weight cycling was significantly associated with an increased risk of adverse health outcomes and was stronger in individuals with normal BMI and females, indicating that these people should be informed about the potential risk of weight cycling.

## Introduction

Obesity is one of the most important health issues that people face, as it is linked to many adverse health outcomes. Many health-conscious people try to lose weight to prevent diseases associated with obesity. In addition to prevention of the various adverse health outcomes directly linked to obesity, many people also try to lose weight due to social pressure, and such attempts are more commonly seen among the young population. Individuals in performance roles such as fashion models, movie stars, and athletes, are also known to lose weight for reasons other than health. For a variety of reasons, an increasing number of people are attempting to lose weight. The prevalence of diet for weight loss in the United States was 7% in men and 14% in women in the 1950–1966 National Health and Nutrition Examination Survey (NHANES) and 40% in men and 57% in women in the 2003–2008 NHANES^1^. The proportion of normal-weight individuals among those who were dieting to lose weight was 20% in men and 46% in women in a 2003–2008 survey in the United States^2^. The prevalence of dieting for weight loss in older people was almost as high as that in younger people. In the 2003–2008 NHANES conducted in the United States, the proportion of people desiring to lose weight was not different between age groups: 59% in men older than 55 years vs. 54% in those younger than 55 years and 70% in women older than 55 years vs. 75% in those younger than 55 years^2^.

Weight loss is often attempted in ways that are not beneficial to health, and even if the initial loss is successful, it is known that most of the weight loss is not maintained. When people fail to maintain their weight loss, the trend is typically to gain back more weight than was lost. This phenomenon is known as body weight fluctuation. As more people attempt to lose weight, the prevalence of body weight fluctuation increases. It is difficult to evaluate the change in the prevalence of body weight fluctuation because the definition of body weight fluctuation has yet to be established. Its range has been estimated to be 20–35% in men and 20–55% in women^1^.

Although earlier studies on fluctuations in body weight, also known as weight cycling, body weight variability, or yoyo dieting, were reported to be associated with increased mortality and morbidity^3–5^, subsequent studies reported inconsistent results^6–8^. This inconsistency may result from various causes, such as body weight change based on self-reported body weights, lack of consensus on the definition of body weight fluctuation (weight cycling vs. weight variability), lack of consideration of intentionality of weight loss, different sample sizes used in the relevant studies, the number and time interval between weight measurements, and a short follow-up period. For this study, weight change was defined in two manners: ^1^ weight cycling was defined as a change in body weight beyond an absolute or relative threshold level^4,9–16^, and (2) weight variability was defined as the standard deviation or mean square error of body weight measurements during the study period^3,4,17,18^.

Stronger and more frequent weight fluctuations may be associated with poorer health outcomes^19^, and the possibility of the threshold effect on the degree and number of weight fluctuations has not been evaluated. Previous studies used average successive variability or absolute amount of change to define body weight fluctuations. Furthermore, most previous studies defined weight fluctuations by self-reported body weight, with questionable accuracy, or by a small number of body weight measurements. Additionally, weight loss in older age can be associated with a larger change in muscle mass than in younger age^20^, and the differential effects according to age have not yet been evaluated.

To determine whether the association between weight cycling and clinical outcomes varied according to the degree and number of weight cycles as well as the baseline age, BMI, and sex, we analyzed nationwide health screening data from healthy adults who underwent health screening five or more times in 2002–2011.

## Results

The baseline characteristics of the participants with BW cycling at 7% or more of BW are presented in Table 1 (refer to the Supplementary Material for other weight cycles). As the number of weight cycles increased, the proportion of males was lowest in the group with two or more weight cycles (Table 1).

**Table 1:** Baseline characteristics of the participants according to the number of BW cycles defined with 7% BW.

	All participants (n = 6,132,569)	Weight cycle = 0 (n = 4,993,335)	Weight cycle = 1 (n = 912,324)	Weight cycle ≥ 2 (n = 226,910)
Men*, n (%)	4,014,871 (65.5)	3,316,300 (66.4)	562,066 (61.6)	136,505 (60.2)
Age*, mean (SD), years	48.96 (12.6)	49.3 (12.6)	48.2 (12.9)	45.6 (12.5)
BMI*, mean (SD), kg/m^2^	23.9 (3.1)	23.9 (3.1)	23.7 (3.2)	23.9 (3.5)
Weight change*, mean (SD), kg	0.95 (4.8)	1.04 (4.8)	0.3 (4.1)	1.7 (6.7)
SBP*, mean (SD), mmHg	122.8 (14.3)	122.9 (14.3)	122.3 (14.5)	121.7 (14.4)
DBP*, mean (SD), mmHg	76.8 (9.8)	76.8 (9.7)	76.5 (9.8)	76.4 (9.9)
FBS*, mean (SD), mg/dL	97.9 (22.7)	97.9 (22.3)	97.7 (24.2)	97.5 (25.9)
TC*, mean (SD), mg/dL	196.0 (36.1)	196.3 (36.0)	194.7 (36.4)	194.5 (36.7)
Smoking status*, n (%)				
Nonsmoker	3,265,979 (53.3)	2,645,159 (53.0)	497,759 (54.6)	123,061 (54.2)
Ex-smoker	1,230,038 (20.1)	1,017,822 (20.4)	170,663 (18.7)	41,553 (18.3)
Current smoker	1,636,553 (26.7)	1,330,355 (26.6)	243,902 (26.7)	62,296 (27.5)
Alcohol consumption*, n (%)				
No	2,880,866 (47.0)	2,324,679 (46.5)	446,941 (49.0)	109,246 (48.1)
< 10 g/day	1,564,561 (25.5)	1,272,243 (25.5)	232,581 (25.5)	59,737 (26.3)
10–19.9 g/day	793,047 (12.9)	654,679 (13.1)	110,624 (12.1)	27,744 (12.2)
20–39.9 g/day	668,007 (10.9)	554,650 (11.1)	90,960 (10.0)	22,397 (9.9)
≥ 40 g/day	226,089 (3.7)	187,085 (3.8)	31,218 (3.4)	7786 (3.5)
Physical activity*, n (%)				
Low (METS < 600)	2,126,966 (34.7)	1,710,386 (34.3)	331,461 (36.3)	85,119 (37.5)
Moderate (600 ≤ METS < 3000)	3,300,415 (53.8)	2,706,988 (54.2)	476,150 (52.2)	117,277 (51.7)
High (METS ≥ 3000)	705,189 (11.5)	575,962 (11.5)	104,713 (11.5)	24,514 (10.8)
History of type 2 diabetes*, n (%)	1,383,120 (22.6)	1,113,671 (22.3)	215,308 (23.6)	54,141 (23.9)
History of hypertension*, n (%)	1,461,935 (23.8)	1,179,850 (23.6)	225,986 (24.8)	56,099 (24.7)
History of heart disease*, n (%)	295,516 (4.8)	241,014 (4.8)	44,294 (4.9)	10,208 (4.5)
History of stroke*, n (%)	181,012 (3.0)	145,165 (2.9)	28,496 (3.1)	7351 (3.2)

*SBP* systolic blood pressure, *DBP* diastolic blood pressure, *FBS* fasting blood sugar, *TC* serum total cholesterol, *BMI* body mass index.

*Indicate p-value < 0.0001 across three groups (weight cycle = 0, 1, or ≥ 2) based on ANOVA and Chi-squared test.

### The association between weight cycling and health outcomes

**Figure 1:**
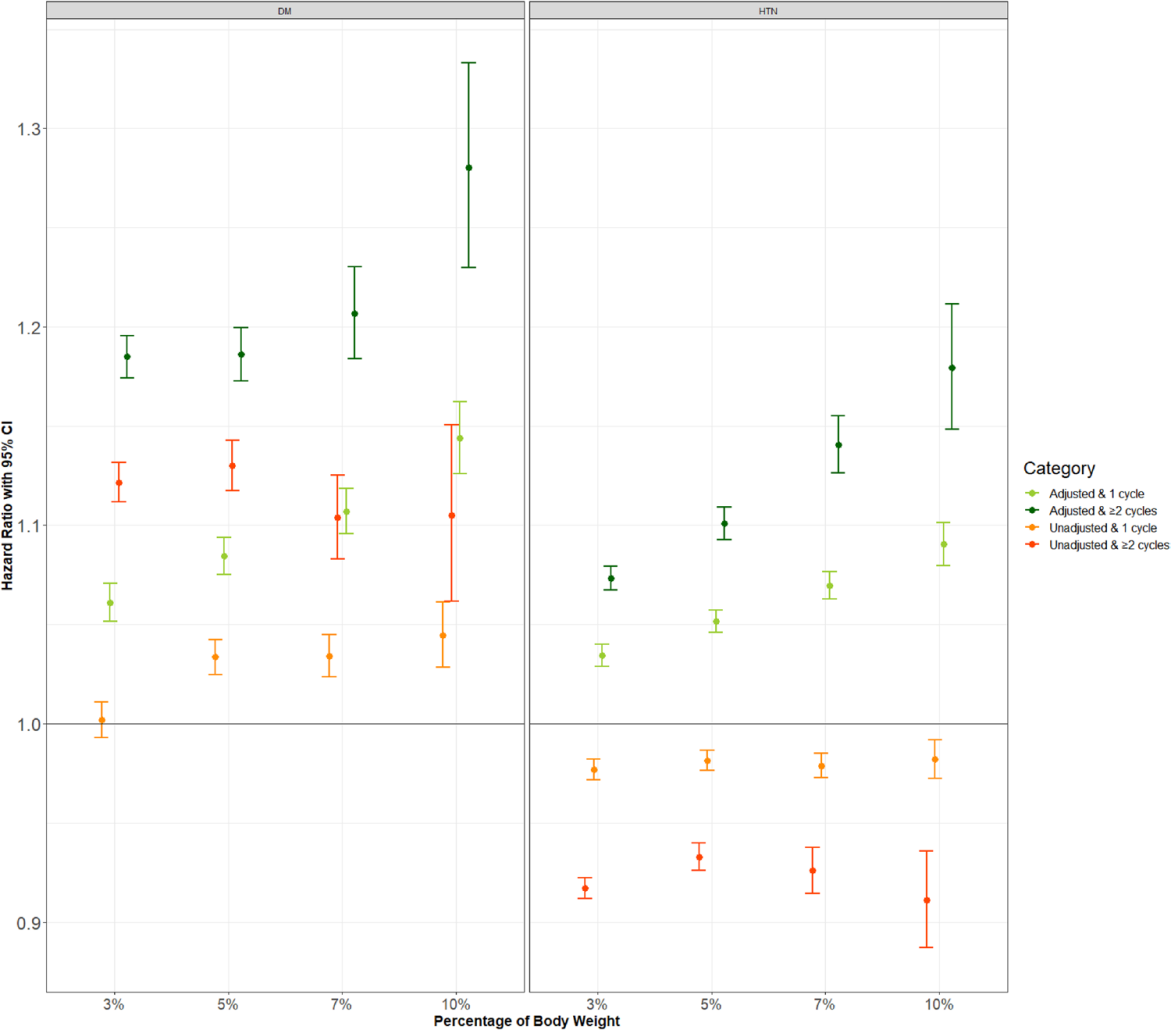
The association between body weight cycling and the risk of type 2 diabetes and hypertension according to various definitions of body weight cycling. Incident diabetes was defined as the prescription of antidiabetic medication or fasting blood sugar ≥ 126 mg/dL. Hypertension was defined as the prescription of hypertensive medication or systolic or diastolic blood pressure ≥ 140/90 mmHg. The hazard ratio was calculated with a Cox proportional hazards regression without adjustment (left, **a**) and with adjustment for age, sex, body mass index, systolic blood pressure, fasting blood sugar, total cholesterol, smoking status, alcohol consumption, physical activity, past medical and family history (right, **b**). Dots and error bars indicate the hazard ratio and 95% confidence interval, respectively.

Weight cycling was associated with an increased risk of incident type 2 diabetes and hypertension. The dose effect of the degree and number of weight cycles was observed as the association increased with the degree and number of weight cycles. The hazard ratios (HRs) of weight cycling for type 2 diabetes and hypertension ranged from 1.053 (95% CI: 1.043–1.063) and 1.037 (1.031–1.042) at one weight cycle of 3% BW to 1.263 (1.213–1.315) and 1.175 (1.144–1.207) at two or more weight cycles of 10% BW. Figure 1 demonstrates how these effects changed for different threshold percentages (3%, 5%, 7%, and 10%) and numbers of weight cycles. These results will be referred to as the main results hereinafter.

### The association between weight cycling and health outcomes according to sex, smoking status, baseline age, and body mass index

**Figure 2:**
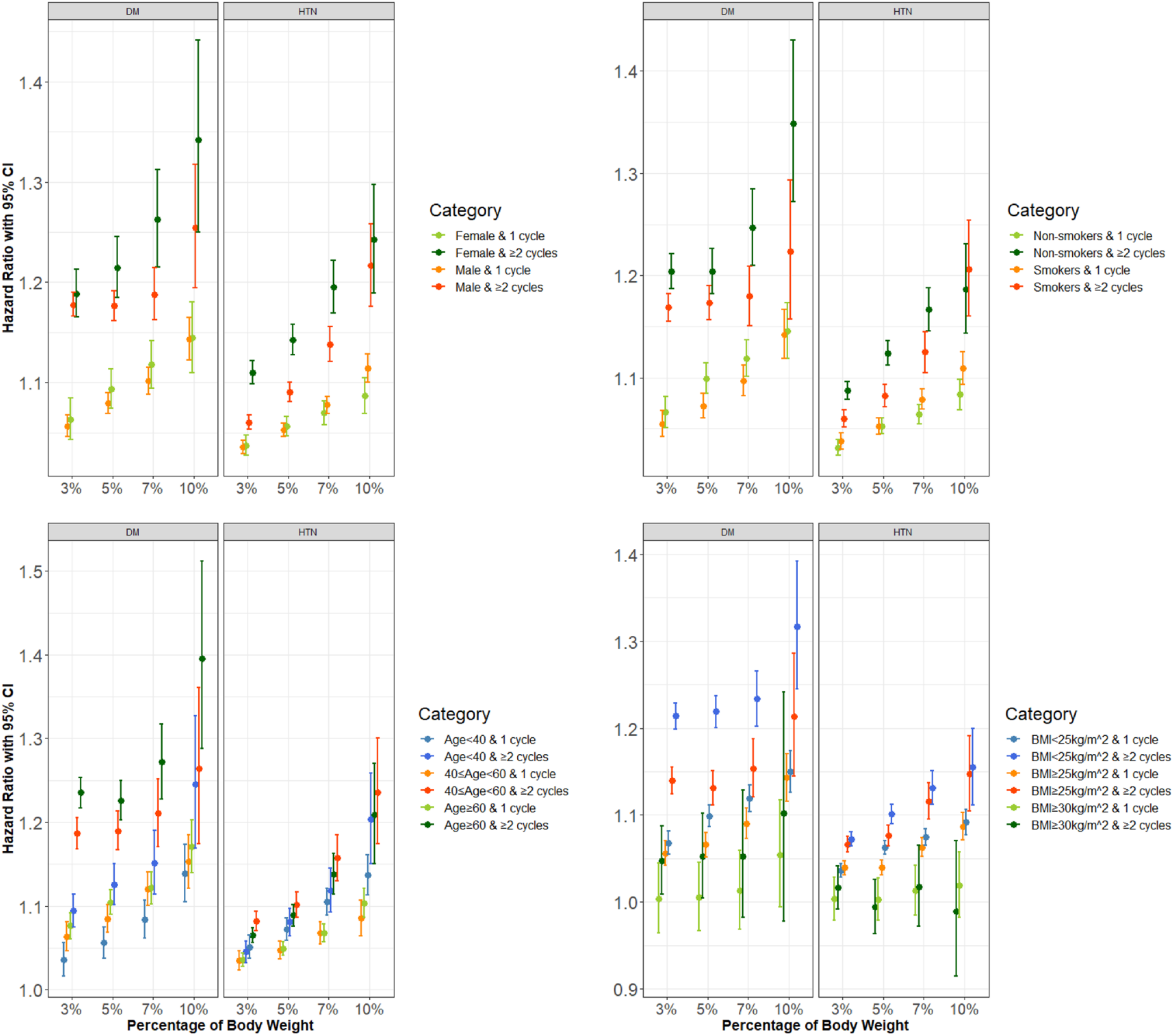
The association between body weight cycling and the risk of type 2 diabetes and hypertension according to sex, smoking status, baseline age, and obesity. Incident diabetes was defined as the prescription of antidiabetic medication or fasting blood sugar ≥ 126 mg/dL. Hypertension was defined as the prescription of hypertensive medication or systolic or diastolic blood pressure ≥ 140/90 mmHg. The hazard ratio was calculated with a Cox proportional hazards regression for different subgroups including sex (top left, **a**), smoking status (top right, **b**), age group (bottom left, **c**), and body mass index (bottom right, **d**) after adjusting for systolic blood pressure, fasting blood sugar, total cholesterol, smoking status, alcohol consumption, physical activity, past medical history, and family history. Dots and error bars indicate the hazard ratio and 95% confidence interval, respectively.

The association between weight cycling and type 2 diabetes followed a dose effect, as in the main results, but was stronger for females than males. The HRs of weight cycling for type 2 diabetes ranged from 1.057 (1.046–1.068) and 1.063 (1.043–1.084) at one weight cycle of 3% BW to 1.255 (1.195–1.317) and 1.342 (1.25–1.442) at two or more weight cycles of 10% BW for males and females, respectively. The association for two or more weight cycles with hypertension was stronger in females than males but was similar for one weight cycle (Fig. 2a).

Smokers and non-smokers subgroups were analyzed separately to investigate the different association under the influence of smoking. Weight cycling for all threshold percentages and numbers showed similar dose effects for type 2 diabetes and hypertension, where non-smokers showed higher HR for type 2 diabetes but not for hypertension (Fig. 2b).

Participants were classified into the young, middle aged, and older groups (< 40 years, ≥ 40 years and < 60 years, and ≥ 60 years). Weight cycling for all threshold percentages and numbers showed similar dose effects for incident type 2 diabetes with stronger effects for older groups. However, the effects of weight cycling on hypertension did not show any clear pattern over increasing threshold percentages and numbers but were still significant factors for hypertension (Fig. 2c). Based on the results, we believe a prospective study in collaboration with family medicine is needed to explore where the cause of weight variability in the older group came from.

Participants were classified according to the baseline BMI (baseline BMI < 25 kg/m^2^ vs. ≥ 25 kg/m^2^), and further BMI groups (BMI ≥ 30 kg/m^2^, BMI ≥ 35 kg/m^2^) were investigated to assess the effect of weight cycling in groups with obesity. For both participants with BMI < 25 kg/m^2^, and BMI ≥ 25 kg/m^2^ weight cycling showed dose effects for incident type 2 diabetes and hypertension, but the effects decreased for the BMI ≥ 25 kg/m^2^ group. The diminishing pattern continued for participants with BMI ≥ 30 kg/m^2^ and BMI ≥ 35 kg/m^2^. For participants with BMI ≥ 30 kg/m^2^, the effects of weight cycles of all thresholds and numbers were insignificant for type 2 diabetes and hypertension except for two or more weight cycles of 5% BW on type 2 diabetes (HR of 1.053, 1.005–1.102). However, all weight cycle effects diminished for both type 2 diabetes and hypertension with BMI ≥ 35 kg/m^2^ (Fig. 2d). Although the commonly accepted idea that individuals who are obese have a higher incidence of type 2 diabetes and hypertension, our study has novel findings. This shows that attention should be paid not only to weight but also to variabilities in weight in the clinical practice.

### The proportional hazard assumption

One important assumption of the Cox proportional hazard is that covariates have multiplicative and constant effects on the hazard (PH assumption). It is important to test whether the PH assumption is satisfied for all variables for the following reasons: ^1^ the association between the variable of interest, weight cycling, and health outcomes may change over time; and (2) after correctly specifying other variables, the association between the variable of interest and health outcome may change. For example, negative hormone receptor status increased the risk of metastases in the beginning but later reduced the risk of metastases^23^.

Schoenfeld residual plots were used to test whether the PH assumption was satisfied for each variable. BMI (categorized), sex, family history of type 2 diabetes and hypertension, and weight cycles were found to violate the PH assumption (Fig. S2). BMI, sex, and family histories were used as strata for the Cox proportional model (Fig. S3-4), and the results were very close to the main results. Furthermore, the Schoenfeld residual plot for the weight cycle showed that beta for weight cycles (3%, 5%, 7%, and 10% alike) increased past t = 3000 days (Fig. S2). This was due to the difference arising from the two groups of people insured by the National Health Insurance.

### Sensitivity analysis

Weight loss after stroke and weight fluctuation after heart disease are widely reported^24^. Therefore, we excluded participants who had a past medical history of stroke or heart diseases (Fig. S3-1). Female participants who did not receive chest X-rays were possibly pregnant at the time of health screening, and there was no available variable indicating pregnancy. Therefore, we excluded women’s health screenings in which chest X-rays were not performed and re-evaluated their weight cycles (Fig. S3-2). Another important factor, waist circumference (WC), was added. WC was a significant factor for type 2 diabetes (1.015, 1.014–1.015) and hypertension (1.019, 1.018–1.019). The effect of weight cycling strengthened further after introducing WC into the model (Fig. S3-3). After adjusting for WC, the HR of weight cycling for type 2 diabetes and hypertension increased for weight cycles of 5%, 7%, and 10% ranging from 1.091 (1.071–1.111) and 1.057 (1.047–1.067) at one weight cycle of 5% BW to 1.446 (1.345–1.554) and 1.287 (1.232–1.344) at two or more weight cycles of 10% BW (Fig. S3-3).

## Discussion

In this study of 6,132,569 participants who underwent five or more health screenings during 2002–2012, we observed that weight cycling was associated with an increased risk of type 2 diabetes and hypertension, with its behavior resembling a dose effect. In subgroup analyses, the association between weight cycling and type 2 diabetes and hypertension decreased as BMI increased. The association between weight cycling and type 2 diabetes increased as age increased, but the association with incident hypertension did not show any trend associated with age.

The dose effect pattern found in the main results did not substantially change after exclusion of the participants who were current smokers and ex-smokers and women who did not undergo chest X-rays to remove the influence of smoking and pregnancy on the weight change.

Despite earlier observations that weight fluctuation was associated with increased morbidity or mortality^3,4^, the subsequent observations were not consistent. Statistically defined weight variability, the standard deviation of BW measurement during the study period or the sum of the relative variability of each BW from the linear regression line of each participant over time has been positively associated with the risk of myocardial infarction and stroke^4^ and the risk of type 2 diabetes^25^. Previous studies have reported a nonsignificant association between weight variability and type 2 diabetes^26^, a positive association between weight variability and metabolic syndrome^27^, and a positive association between statistically defined weight variability and all-cause and cause-specific mortality^16^. Because increased weight variability may be caused not only by repeated cycles of weight reduction and regain but also by continuous weight reduction or gain, weight cycling, defined as weight change beyond a certain level, may be a more adequate definition of repeated cycles of weight reduction and regain.

Weight cycling was reported to be positively^4,19^ and negatively^10^ associated with the risk of type 2 diabetes, positively^11,28^ with the risk of hypertension, and positively^4^ with the risk of myocardial infarction. Some studies reported no significant association between weight cycling and blood pressure^29^. Weight cycling has been positively associated with all-cause or cause-specific mortality^16^. A nonsignificant association between weight cycling and mortality has also been reported^15,30^. Field et al. defined severe and mild weight cycling with both the amount and number of BW changes and observed no significant association between BW fluctuation and all-cause mortality and the risk of hypertension and type 2 diabetes^12–14^. Previous studies defined weight cycling with arbitrarily set criteria. No study to date has evaluated various criteria levels and tried to find any threshold level where the risk of health outcomes began to increase. To the best of our knowledge, our study is the first to report the dose effect response of the degree and the number of weight cycles in its association with health outcomes.

The intentionality of weight reduction or the accuracy of recalled BW could influence the observed association between weight change and health outcomes^12–14^. Although the recalled BW was reported to have a good correlation with the actual BW in general^31^, it might not be possible to exclude the possibility that the change in BW might be exaggerated by the participants who experienced a BW cycle caused by intentional weight reduction. Moreover, in addition to these two factors, the insufficient size of the sample, small number of actual BW measurements, insufficient follow-up duration, and undiagnosed health outcomes such as type 2 diabetes or hypertension may be other limitations of previous studies. Although a nonsignificant association of intentional weight cycling three or more times was reported, the ascertainment of weight cycling and morbidity was based on self-report in those studies^12–14^.

In our study, more than 6 million participants selected from the nationwide registry, with 5 or more actual BW measurements, relatively long observation periods (10 years of BW measurement and an additional 8 years of observation), and health outcomes identified not only by health insurance claims data but also by health screening data, can overcome the limitations of previous studies. The number of weight cycles was also considered in this study, and weight cycling of two or more times was associated with a higher risk of health outcomes than weight cycling once. Although intentionality was not evaluated in this study, it may be practically resolved because unintentional weight reduction due to diseases is unlikely to be associated with weight cycling of two or more times.

Repeated cycles of smoking and abstinence may cause both weight cycling and an increased risk of health outcomes^32^. In this study, nonsmokers were investigated separately and found to have stronger association patterns between weight cycling and health outcomes. In women, pregnancy may cause substantial weight change^33^. After excluding health screenings of female participants without chest X-ray, the results did not change significantly.

In this study, varying definitions of weight cycling were evaluated, such as 3%, 5%, 7%, and 10% BW. The risk of type 2 diabetes was greater for the older population, but that of hypertension was not. The former may be due to the limited recovery of lost muscle mass during the weight reduction in this age group compared to the younger population^20,21^. The association between weight cycling and health outcomes was more significant in the participants with normal baseline BMI than in those with BMI ≥ 25, ≥ 30, or ≥ 35 kg/m^2^. This observation is in line with the findings that lean people have higher gain in body fat mass after weight loss followed by weight gain^33^ and has important clinical implications: unnecessary weight reduction in the population without obesity, for example, weight reduction for cosmetic purposes, may be more harmful than in the population with obesity. This observation needs to be confirmed in future studies.

Despite its strengths, this study has several limitations. First, the rapidity of weight change was not evaluated. It was possible that more rapid weight cycling caused a more severe impact on physiology. Second, the interval between adjacent health screenings was one or two years, and the weight cycling between two adjacent health screenings could not be captured. This failure to capture weight cycling between the health screenings might bias the results to null. Third, the health outcomes observed in this study were not confirmed by physicians. Finally, the results of this study were observed in the same ethnic population. Therefore, generalization of the results of this study should be done with caution.

In a study of more than 6 million adults who underwent five or more health screenings, weight cycling was found to have positive associations with negative health outcomes, with stronger associations as the degree and number of weight cycles increased. Weight cycling was associated with an increased risk of adverse health outcomes in the nonobese population. Moreover, an increased risk of type 2 diabetes for older individuals was associated with weight cycling. Therefore, weight reduction, especially in individuals who were nonobese or older, should be carried out cautiously, and not only the risk of absolute weight but the risk of repeated weight cycles should be taken into consideration.

## Research design and methods

### Study population

All citizens in Korea are covered by a single health insurance operated by the National Health Insurance (NHIS) and must undergo mandatory regular health screening at designated hospitals every year or every two years. The NHIS collects all health screening data from designated hospitals. The details of health screenings, such as eligibility criteria, participation rate, and collected data, have been reported previously^21^. This study was based on the National Health Information Database of the NHIS containing health insurance claims data and health screening data. The health screening data consisted of two parts: ^1^ lifestyle factors, such as alcohol intake, physical activity, smoking, past medical history, current medical conditions, and family history, which were collected by a common questionnaire completed by the participants; and (2) measurement data, such as height, weight, and blood pressure, and laboratory test results including blood hemoglobin, fasting blood sugar, liver function test, blood lipids, serum creatinine, and urine protein by urine dipstick and chest X-ray results.

The study is in accordance with relevant guidelines and regulations. The institutional review board of Seoul National University Hospital approved this study (IRB number: 2001-077-1094) and waived the requirement to obtain any informed consent because the data provided by the NHIS were anonymized in compliance with the confidentiality guidelines.

Between January 2002 and December 2011, a total of 6,358,895 participants aged 20 or older underwent at least five health screenings. Participants with no follow-up screening between January 2012 and December 2019 (11,078 participants) were excluded. Furthermore, 215,248 participants with missing variables at baseline were excluded. The remaining 6,132,569 participants were followed from January 2012 to December 2019 (Fig. S1). The information about participant’s nationality is not provided in the screening and the ratio of foreign nationals residing in Korea during the period is less than 2% and official statistics on ethnicity are not released. Therefore, ethnicity and nationality were not included in this study.

### Clinical definitions

Smoking status was classified as nonsmokers, ex-smokers, and current smokers. Alcohol consumption was categorized as no consumption, < 10 g/day, 10–19.9 g/day, 20–39.9 g/day, and ≥ 40 g/day. Physical activity was assessed using the long-form International Physical Activity Questionnaire (IPAQ), where participants are classified based on metabolic equivalents (METS) values of < 600 METS, 600–3000 METS, and ≥ 3000 METS^22^.

**Figure 3:**
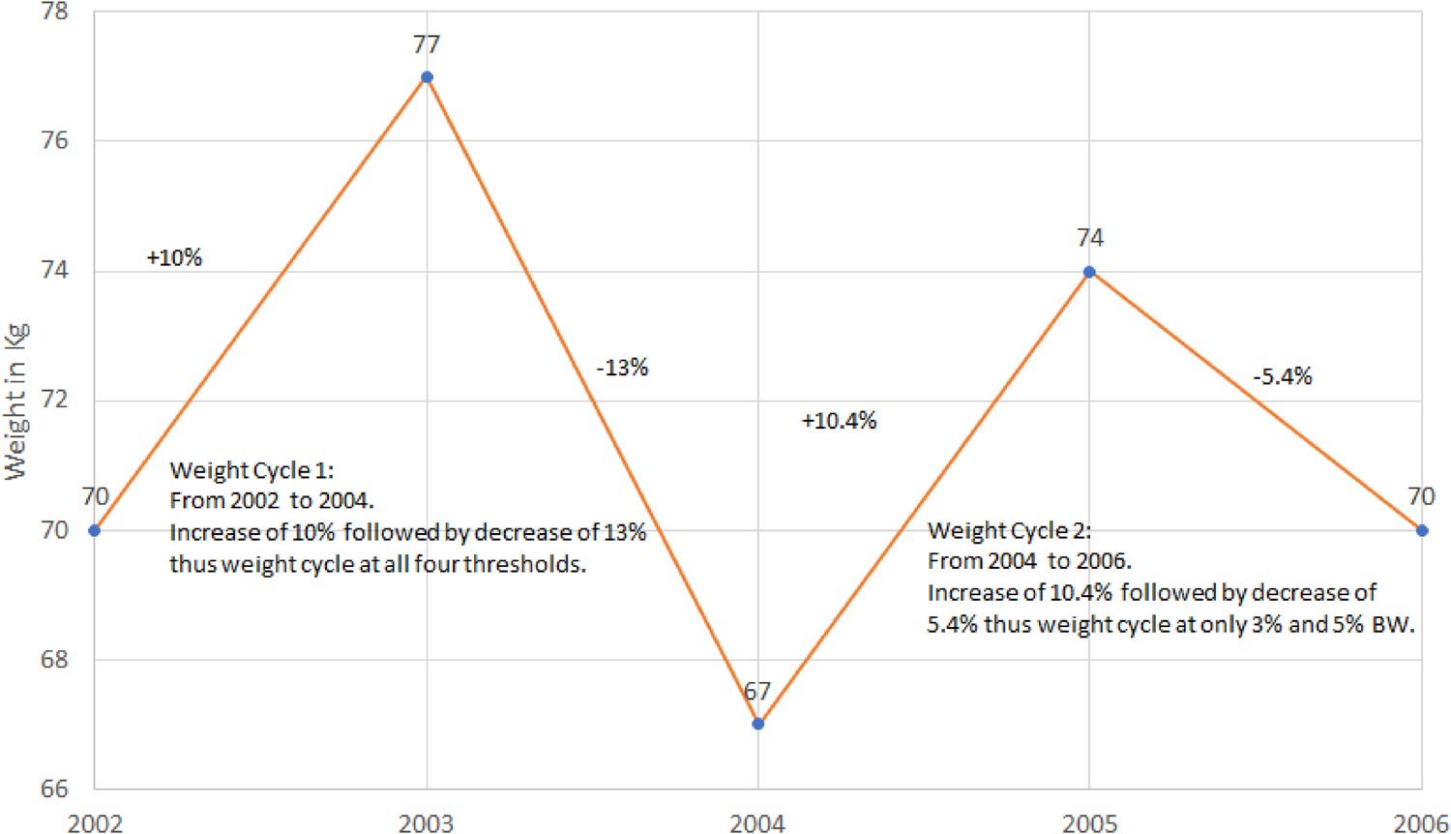
Example of body weight cycling for a participant with 5 measurements with 2 cycles at 3% and 5% thresholds, and 1 cycle at 7% and 10% thresholds. The BW cycle was defined by identifying measurements where the BW changed more than the specified threshold percentage of BW. The following example illustrates how such cycles were identified at various threshold percentages.

Body weight (BW) cycling was defined with multiple thresholds (3% or more, 5% or more, 7% or more, and 10% or more of BW) to study the association between BW cycling and type 2 diabetes and hypertension. First, all BW measurements were scanned to identify the first two measurements where BW change was equal to or greater than the threshold. Then, other BW measurements were scanned to locate the point where a BW change of the threshold percentage or greater occurred in the opposite direction. This completed one BW cycle at the threshold percentage, and the search for the next BW cycle began at the measurement where one cycle ended. Example of identification of a weight cycle is illustrated in Fig. 3.

The primary outcomes were incident type 2 diabetes or hypertension observed between January 2012 and December 2019. Incident type 2 diabetes mellitus was defined as the prescription of antidiabetic medication in the health insurance claim data or as fasting blood sugar ≥ 126 mg/dL in the health screening data. Hypertension was defined as the prescription of antihypertensive medication in the health insurance claim data or as systolic or diastolic blood pressure ≥ 140/90 mmHg in the health screening data. Participants with prevalent type 2 diabetes and hypertension or prescriptions of relevant drugs on or before Dec 31, 2011, were excluded when the risk of incident type 2 diabetes or hypertension was estimated.

### Statistical analysis

Continuous variables are presented as the mean ± standard deviation (SD), and categorical variables are presented as frequencies and percentages. The three groups of participants (those with 0, 1, or ≥ 2 weight cycles) were compared using one-way ANOVA for continuous variables and the chi-square test for categorical variables. The association between weight cycling and outcomes was evaluated with a Cox proportional hazard model to estimate the hazard ratios (HRs) and 95% confidence intervals (CIs) adjusted by confounding variables at baseline: age, sex, body mass index (BMI), systolic/diastolic blood pressures, fasting blood sugar, total cholesterol, smoking status, alcohol consumption, physical activity, and medical history (hypertension, type 2 diabetes mellitus, heart disease, and stroke). The proportional hazard is a key assumption of the Cox model, and violation of this assumption leads to misleading results. The proportionality test is a chi-square-based test, which rejects even a small deviation when the sample size is sufficiently large. For this reason, the proportional hazard assumption was visually tested with Schoenfeld residual plots in addition to the proportionality test. The effects of weight cycling after stratifying for variables violating the PH assumption were still consistent with the main results presented in this paper.

In addition, subgroup analyses were conducted by the baseline age groups (< 40 years, 40–59 years, ≥ 60 years) and baseline BMI (< 25 kg/m^2^, ≥ 25 kg/m^2^, ≥ 30 kg/m^2^, ≥ 35 kg/m^2^). For sensitivity analysis, subgroup analyses were performed. Participants who received health screening every year or every two years were analyzed to investigate whether weight cycling effects differed in the two groups. Furthermore, participants who were current smokers or ex-smokers and women who did not undergo chest X-rays were excluded to remove the influence of smoking and pregnancy on BW changes.

Two-tailed *P* values < 0.05 were considered statistically significant. All statistical analyses were performed using R 4.0.0 (R Core Team, Vienna, Austria) and SAS version 9.4 (SAS Institute, Cary, NC).

## Supplementary Information

Supplementary Figures.
