# Supplementary material for: The association between diabetes and hypertension with the number and extent of weight cycles determined from 6 million participants
Source: Sci Rep. 2022 Mar 28;12:5235. doi: 10.1038/s41598-022-09221-w (PMC8960790; doi:10.1038/s41598-022-09221-w)
Supplement: Supplementary file 1 — Supplementary Figures. [file 41598_2022_9221_MOESM1_ESM.docx]

**Supplementary figure legends**

**Fig. S1. The flow diagram of study population**


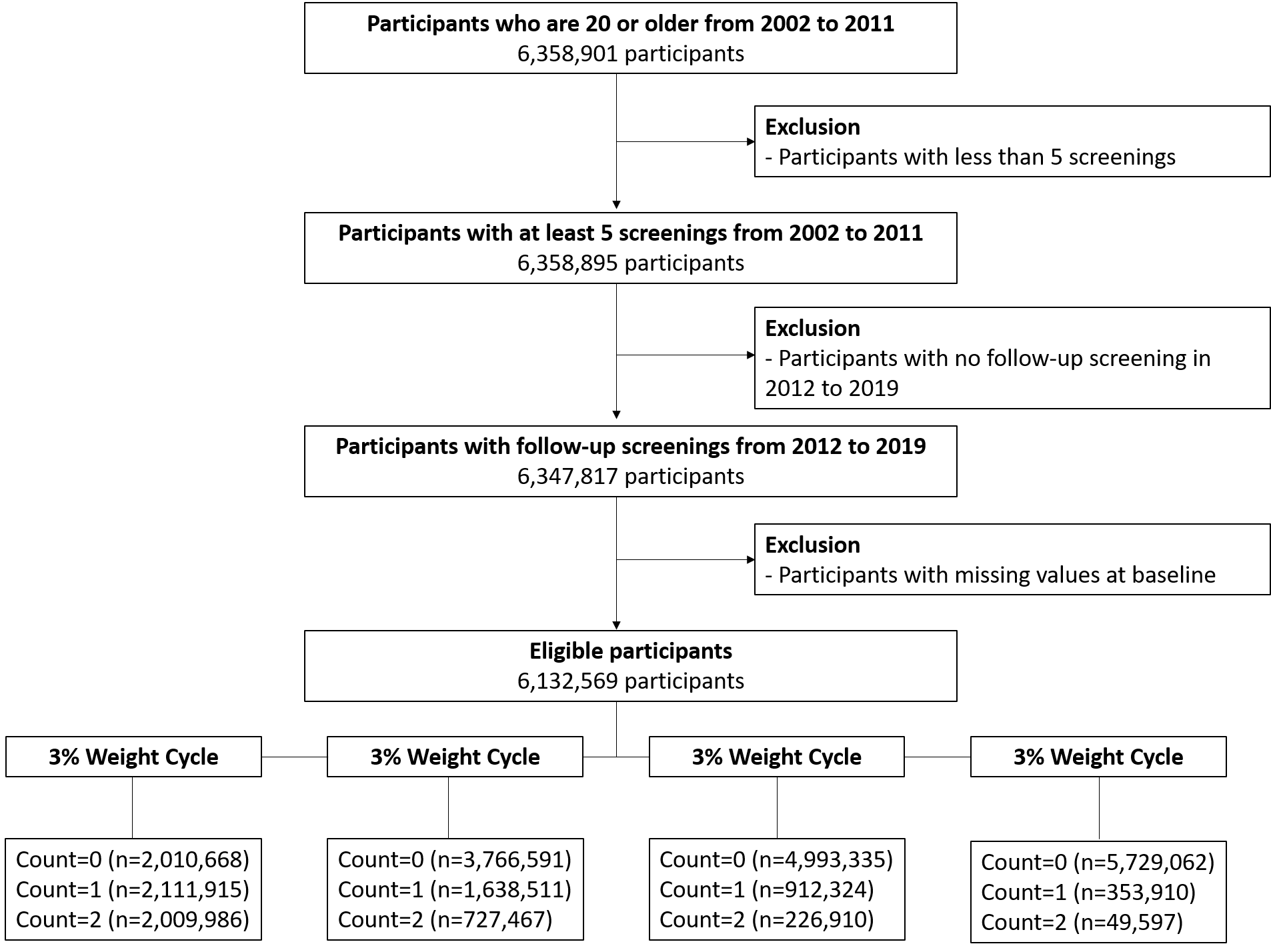


**Fig. S2. The Schoenfeld residual plot for weight cycling.**

The Schoenfeld residual plot for weight cycling for both type 2 diabetes and hypertension showed deviation from a random pattern, indicating violation of the proportional hazard assumption (Supplementary Figure 2-3).


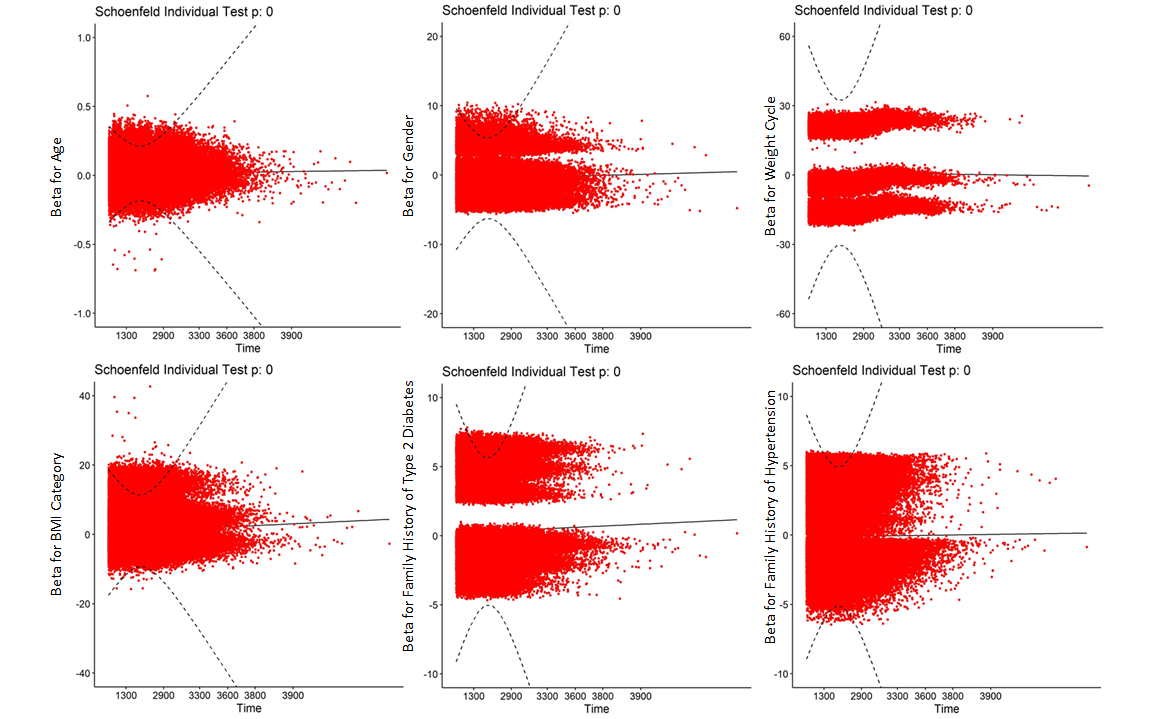


**Fig. S3. The results of sensitivity analyses.**


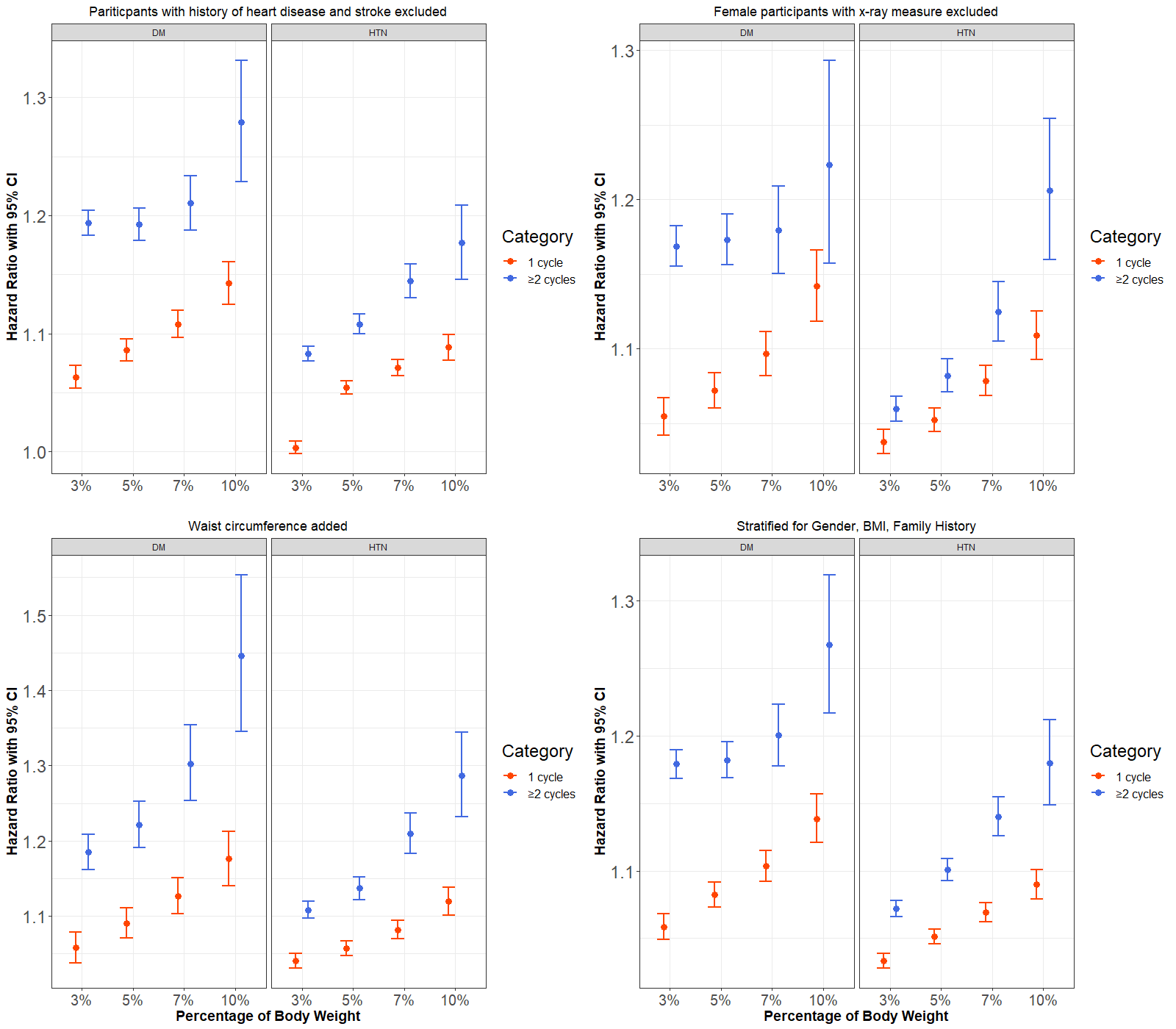


The association between body weight cycling and type 2 diabetes and hypertension was assessed after removing participants with history of heart disease or stroke (Top left, Supplementary Figure 3-1) and female participants who underwent chest X-ray (Top right, Supplementary Figure 3-2). The association between body weight cycling and type 2 diabetes and hypertension was assessed after adjusting for waist circumference (Bottom left, Supplementary Figure 3-3) and stratifying for variables that violate the PH assumption, such as sex, BMI, family history of type 2 diabetes and hypertension (Bottom right, Supplementary Figure 3-4).
